# Influence of Climate on Disease

**Published:** 1874-09-01

**Authors:** 

**Affiliations:** Prytania St., New Orleans


### INFLUENCE OF CLIMATE ON DISEASE.

MESSRS. EDITORS: I propose to follow a former letter to your journal by a cursory account of some of the acute diseases which I have treated in this city.

The advantage to accrue from accumulating medical observations taken in different latitudes and climates, rests wholly upon the proposition that climatic conditions affect the clinical career of diseases. It is now some years since Boudin published a paper in the "Annales d'Hygiene Publique," entitled "Recherches sur l'Acclimatement des Races Humaines sur divers points du Globe." This article commences with this enquiry: "Is man the cosmopolite he has heretofore esteemed himself to be, or do the preservation of his life and the propagation of his species depend upon climatic conditions bearing more or less close relation to those of the land of his birth?" Whatever postulate the well man may choose to assume in regard to his capabilities to support or enjoy life, in any and all climates, the physician who changes his latitude, or who serves a community whose pursuits require exposure in different latitudes, soon learns how important it is for his success, to obtain the cue to those particular manifestations which are due to climate or race.

It is a fact, almost universal to our profession, that when acute inflammations become a topic of discussion,

"pneumonia" is selected as affording the best examples for illustration. Aside from the force of professional custom, as determining a precedent, there are, in my opinion, other and more important reasons why the study of this disease essentially bears upon questions connected with climatic influences upon acute disorders.

Three propositions may be stated which cover the whole ground. The first of these holds that in warm climates certain morbific agents, by their greater abundance or activity, increase the liability to pneumonia. While it is generally admitted that exposure to low thermometric readings is the most prolific cause of pneumonia in cold latitudes, it must be remembered that falls of temperature, relatively as sudden and considerable, likewise occur in this latitude. Pneumonia is, consequently, with us, a disease mostly prevalent during the cooler half of the year. Prof. Chaille's compilation of mortality statistics of New Orleans, shows that in the five years ending with 1870, there occurred 1028 deaths from pneumonia during the half years ending with April, against 617 during the half years ending with October.

But in addition to variations of temperature, there is diffused over every square mile bordering the lower third of the Mississippi, an agent more mischievous against human health and life than all others combined. This is malarial poison. Does it increase liability to attacks of pneumonia? I add my opinion most emphatically to that of the learned Greisinger, that it does. This opinion rests both upon observation and analogy. The proofs furnished by observation I will not undertake to lay before your readers, as they are too voluminous for mere desultory letters. The proofs afforded by analogy, may be found in that close resemblance in blood changes, between the malarial cachexia and the cachexia of Bright's disease. All of us admit that Bright's disease increases the frequency of attacks of pneumonia in common with inflammations of other surfaces.

The second of these propositions holds that the morbific agents, alluded to in the first proposition, exert a very unfavorable effect upon the mortality of pneumonia occurring during their presence in the system. Whatever discussions may be considered proper before accepting or rejecting the first proposition, the truth of the second cannot be questioned. The degree to which one of these agents is capable of influencing the death rate of pneumonia, will be shown as we progress.

The third proposition is, that long-continued exposure to the heat of warm climates, by diminishing constitutional vigor, renders pneumonia a more fatal disease in warm climates than in those which are colder and more bracing. All physicians know that the functional lesions of pneumonia entail upon the human economy, an amount and variety of derangement of chemistry which scarcely any other disease can inflict. It thus becomes a most extraordinary test of the constitutional vigor of its subjects.

However our short-comings may be attempted to be explained, it is certainly true that we invariably fail in obtaining such gratifying results in the treatment of pneumonia, as those which Bennett boasts of in Edinburgh, or of the surgeon in the British army while stationed at Montreal.

Prof. Chaille shows that the proportion of deaths to cases treated in Charity Hospital, during ten years, was two in five. These figures relate to the whole hospital, and cannot, therefore, be chargeable to any individual imperfections of treatment. Again, taking my own hospital service for seven half years, beginning October 1st and ending April 1st, and the proportion of deaths reduced to Prof. Chaille's rule of estimate, was a little under one and three-fifths for each five cases. We had, however, no selection of cases, and those brought in when moribund are included in the count.

The most common and serious complication was malaria. This is recognized to be so universally present, that I never begin the treatment of a case of pneumonia without administering free and repeated doses of quinine, immediately succeeding the action of a mercurial or saline purge, if such medication was demanded. I gave from a scruple to half a drachm in doses of five to ten grains every third hour. If pain was a prominent symptom, or even if it were not present, and there was no contra-indication, I combined five grains of Dover's powder, or its equivalent in some other form, with each dose of the quinine. The contra-indication to opium in pneumonia in this climate, is the bronchorrhœa and pulmonary œdema often present. Blisters I very seldom resorted to. More often I used, for urgent pain, a hot turpentine stupe. In every case I kept the chest continually enveloped with a sheet of oiled muslin, under which was neatly applied a band of flannel preferably wrung from warm water before its application. For excessive fever I used digitalis, veratrum viride, or aconite. Patients were encouraged to drink largely of lemonade, generally made of a diluted infusion of flax-seed instead of water. If I desired a gentle effect upon the bowels, a teaspoonful of bitartrate of potash was used to each tumbler of the lemonade. The patient's nutrition was made a point of special attention, and all adynamic symptoms were met by concentrated diet and alcoholic stimulants. Carbonate of ammonia was much prescribed. I was never able to satisfy myself that either the carbonate or the acetated solution possess the virtues ascribed by Dr. Chambers. In two cases death was hastened, if not principally produced, by heart-clot, which was diagnosed, in one instance, twenty hours before death. Large doses of carbonate of ammonia were given, for at that date Richardson's doctrines were neither recanted nor disputed. No apparent benefit resulted.

I have never been able, practically, to verify the statements made by some writers in regard to the occurrence of *intermittent* acute inflammations. While, without violence to known pathological laws, we admit that the revulsion of a malarial paroxysm is quite sure to aggravate any co-existing inflammation, no case has occurred to me in which I was able to perceive that the progress of the inflammation was limited to the malarial paroxysm.

I have always observed the strictest watchfulness in order to prevent the recurrence of malarial paroxysms during the progress of pneumonia, or during convalescence from it. Any increase of the discrepancy between morning and evening temperature would furnish an indication for quinine. Even if this did not exhibit such indications of waxing malarial influence, I generally considered it a good rule to give from ten to fifteen grains of quinine every third day.

In looking over my private practice for three years, I find that I have treated ten cases of pneumonia, one of which resulted fatally. Three of this number were of the negro race, and the fatal case belonged to this group. The negro's constitution succumbs to attacks of acute disease more readily than the white man's, and this is especially true in regard to pneumonia. But as this letter has already reached greater length than designed, I will leave the discussion of this point to a future number.

Respectfully,

MEDICUS MERIDIONALIS,

Prytania St., New Orleans, July 20th, 1874.

